# A new combination and taxonomic notes in *Pseudobombax* Dugand (Malvaceae)

**DOI:** 10.3897/phytokeys.85.13930

**Published:** 2017-08-09

**Authors:** Jefferson G. Carvalho-Sobrinho, Laurence J. Dorr

**Affiliations:** 1 Universidade Federal do Vale do São Francisco – UNIVASF, Colegiado de Ciências Biológicas, BR-407, Km 12, Petrolina, Pernambuco, 56300-990, Brazil; 2 Department of Botany, National Museum of Natural History, MRC-166, Smithsonian Institution, P.O. Box 37012, Washington, D.C. 20013-7012, U.S.A.

**Keywords:** Bombacoideae, *Bombax*, Brazilian Flora 2020, nomenclature, synonymy

## Abstract

Taxonomic notes in the Neotropical genus *Pseudobombax* (Malvaceae) are presented. One new combination and two new heterotypic synonyms for taxa originally described from Brazil and Ecuador are proposed based on both morphological and previously published molecular evidence. The taxonomic changes will be adopted in a treatment of *Pseudobombax* for the upcoming Brazilian Flora 2020 and in a forthcoming revision of the genus throughout its range.

## Introduction

Field and herbarium studies of the Neotropical genus *Pseudobombax* Dugand (Malvaceae) along with previously published molecular phylogenetic studies (Carvalho-Sobrinho et al. 2016) revealed that taxonomic changes are needed in the genus. Nomenclatural changes are proposed herein for taxa that will be included in a monograph of *Pseudobombax* for Brazil (Carvalho-Sobrinho in prep.), which is being prepared for the upcoming Brazilian Flora 2020 (http://floradobrasil.jbrj.gov.br/). The changes also will be incorporated in a taxonomic revision of the genus throughout its range.

## Methods

Taxonomic literature related to *Pseudobombax* was evaluated in order to check typifications and synonymies of taxa. Information about type specimens was derived from protologues and checked against major online nomenclatural indices (Tropicos – http://www.tropicos.org/; JSTOR Global Plants – https://plants.jstor.org). Herbarium abbreviations follow *Index Herbariorum* (Thiers 2017). Type specimens that have been examined in person by the first author are followed by exclamation marks. When digital images were discovered online, a barcode number is given for the specimen followed by its source.

## Nomenclatural changes

### Pseudobombax majus

Taxon classificationPlantaeMalvalesMalvaceae

(A. Robyns) Carv.-Sobr., comb. & stat. nov.

urn:lsid:ipni.org:names:60474975-2

Basionym. Pseudobombax grandiflorum var. majus A. Robyns, Bull. Jard. Bot. État Bruxelles 33(1): 56. 1963. — Type: Brazil. Minas Gerais: Viçosa, Chacha Valley, near Chacha House, 17 May 1930, *Y. Mexia 4711* (holotype: F! [F neg. 57390, F0052177F, JSTOR image]; isotypes: BM [BM000645666, JSTOR image], G!, K! [K000913925, JSTOR image], MO! [MO-357466, JSTOR image], NY! [NY133608, JSTOR image], P! [P06622849, JSTOR image], S! [S10-38043], U [U0000789, JSTOR image], US! [US00101966, JSTOR image], VIC!, WIS!).

#### Notes.

Robyns (1963) proposed Pseudobombax grandiflorum var. majus based on specimens with longer pedicels and staminal tubes as well as wider calyces and petals than are found in the nominate variety. Analysis of the protologue and type material of this variety and the nominate one, along with a comprehensive examination of herbarium specimens, reveals that P. grandiflorum var. majus is also morphologically distinct from the nominate variety in having mostly 9–11 (vs. mostly 5) leaflets (7 leaflets rarely occur in both taxa), often 3–7-flowered cymes (vs. flowers solitary, rarely 2–3-flowered cymes), and 5-angulate (vs. 5-costate) fruits in cross-section. Moreover, P. grandiflorum var. majus has calyces that are urceolate (vs. cupuliform) and often lobed (vs. truncate).

In addition to these morphological differences, the two taxa can be distinguished by their distribution in Eastern Brazil. Pseudobombax grandiflorum var. majus inhabits semi-deciduous forests and occasionally granitic outcrops in wet forests whereas the nominate variety inhabits mainly coastal restinga vegetation. Thus, morphological evidence along with the parapatric distribution are sufficient to recognize Pseudobombax grandiflorum var. majus at species rank.

*Pseudobombax majus* can be distinguished from its sister species *P. longiflorum* (Mart.) A. Robyns (Carvalho-Sobrinho et al. 2016: fig. 2), a widespread species in South American savannas, by its cuneate (vs. truncate) leaflets, shorter (c. 10 vs. 16–50 mm long) petiolules, and its 5-angulate (vs. circular) fruits in cross-section.

### Pseudobombax millei

Taxon classificationPlantaeMalvalesMalvaceae

(Standl.) A. Robyns, Bull. Jard. Bot. État Bruxelles 33: 69. 1963.

Basionym. Bombax millei Standl., Trop. Woods 45: 16. 1936. — Type: Ecuador. Sept 1929, *L. Mille 868* (holotype: F! [F0052111F, F0052112F, JSTOR image]). = Pseudobombax guayasense A. Robyns, Bull. Jard. Bot. État Bruxelles 33(1): 68. 1963. — Type: Ecuador. Guayas: vicinity of Guayaquil, Cerro Azul, 10 Sept 1955, *E. Asplund 17588* (holotype: S! [S10-39074, S10-39076, S-R-11308, JSTOR image]!; isotypes: BR [BR0000006961367, JSTOR image], NY! [NY00133609, JSTOR image]), **syn. nov.**

#### Notes.

The characters (leaflet shape, calyx dimensions, and pubescence of staminal tubes) used by Robyns (1963) to segregate *Pseudobombax guayasense* are not sufficient to distinguish it from *P. millei* based on a more comprehensive sampling of herbarium collections. Previously, phylogenetic analyses of DNA sequences were interpreted as supporting the accessions examined of these two taxa as sister groups (Carvalho-Sobrinho et al. 2016: fig. 2), but we now consider *P. guayasense* to be a synonym of *P. millei* on the basis of combined morphological and molecular evidence.

### Pseudobombax munguba

Taxon classificationPlantaeMalvalesMalvaceae

(Mart.) Dugand, Mutisia 9: 4. 1952.

Basionym. Bombax munguba Mart., Nov. Gen. Sp. Pl. 1: 93, t. 99. 1826. — Type: Brazil. Amazonas: Rio Negro, *Martius s.n.* (lectotype, designated by Robyns (1963): M! [M0211657, JSTOR image]). = Pseudobombax amapaense A. Robyns, Mem. New York Bot. Gard. 17(1): 195. 1967. — Type: Brazil. Amapá: Rio Jari, near Cachoeira Miriti, 0°41'N, 53°6'W, 180 m, 25 Aug 1961, *W. A. Egler & H. S. Irwin 46673* (NY! [NY00133605, NY00133606, NY00133607, JSTOR image]), **syn. nov.**

#### Notes.

The characters (tree height and pubescence of staminal tubes) used by Robyns (1967) to segregate *P. amapaense* do not permit it to be distinguished from *P. munguba* based on a more comprehensive sampling in the field and in herbarium collections. Previously, phylogenetic analyses of DNA sequences were interpreted as supporting the accessions examined of these two taxa as sister groups (Carvalho-Sobrinho et al. 2016: fig. 2), but we now consider *P. amapaense* to be a synonym of *P. munguba* on the basis of combined morphological and molecular evidence.

## Supplementary Material

XML Treatment for Pseudobombax majus

XML Treatment for Pseudobombax millei

XML Treatment for Pseudobombax munguba
